# Participatory Women's Groups with Cash Transfers Can Increase Dietary Diversity and Micronutrient Adequacy during Pregnancy, whereas Women's Groups with Food Transfers Can Increase Equity in Intrahousehold Energy Allocation

**DOI:** 10.1093/jn/nxy109

**Published:** 2018-07-20

**Authors:** Helen A Harris-Fry, Puskar Paudel, Tom Harrisson, Niva Shrestha, Sonali Jha, B James Beard, Andrew Copas, Bhim P Shrestha, Dharma S Manandhar, Anthony M de L Costello, Mario Cortina-Borja, Naomi M Saville

**Affiliations:** 1 London School of Hygiene & Tropical Medicine, London, United Kingdom; 2 Mother and Infant Research Activities (MIRA), Kathmandu, Nepal; 3 Institute for Global Health; 4 Great Ormond Street Institute of Child Health, University College London, London, United Kingdom

**Keywords:** pregnancy, dietary adequacy, nutrition, food allocation, supplements, cash transfers, women's groups, community interventions, Nepal, maternal health

## Abstract

**Background:**

There is scarce evidence on the impacts of food transfers, cash transfers, or women's groups on food sharing, dietary intakes, or nutrition during pregnancy, when nutritional needs are elevated.

**Objective:**

This study measured the effects of 3 pregnancy-focused nutrition interventions on intrahousehold food allocation, dietary adequacy, and maternal nutritional status in Nepal.

**Methods:**

Interventions tested in a cluster-randomized controlled trial (ISRCTN 75964374) were “Participatory Learning and Action” (PLA) monthly women's groups, PLA with transfers of 10 kg fortified flour (“Super Cereal”), and PLA plus transfers of 750 Nepalese rupees (∼US$7.5) to pregnant women. Control clusters received usual government services. Primary outcomes were Relative Dietary Energy Adequacy Ratios (RDEARs) between pregnant women and male household heads and pregnant women and their mothers-in-law. Diets were measured by repeated 24-h dietary recalls.

**Results:**

Relative to control, RDEARs between pregnant women and their mothers-in-law were 12% higher in the PLA plus food arm (log-RDEAR coefficient = 0.12; 95% CI: 0.02, 0.21; *P *= 0.014), but 10% lower in the PLA-only arm between pregnant women and male household heads (−0.11; 95% CI: −0.19, −0.02; *P *= 0.020). In all interventions, pregnant women's energy intakes did not improve, but odds of pregnant women consuming iron-folate supplements were 2.5–4.6 times higher, odds of pregnant women consuming more animal-source foods than the household head were 1.7–2.4 times higher, and midupper arm circumference was higher relative to control. Dietary diversity was 0.4 food groups higher in the PLA plus cash arm than in the control arm.

**Conclusions:**

All interventions improved maternal diets and nutritional status in pregnancy. PLA women's groups with food transfers increased equity in energy allocation, whereas PLA with cash improved dietary diversity. PLA alone improved diets, but effects were mixed. Scale-up of these interventions in marginalized populations is a policy option, but researchers should find ways to increase adherence to interventions. This trial was registered at www.controlled-trials.com as ISRCTN 75964374.

## Introduction

The nutritional status of South Asian populations is among the poorest in the world (1, 2), but there is a striking lack of evidence from this region on the effectiveness of common nutrition interventions (3). Interventions such as food supplements, food vouchers, and cash transfers could plausibly supplement inadequate South Asian diets (4). In turn, this could break the intergenerational cycle of undernutrition, particularly if targeted to pregnant women, because underweight women are 64% more likely to have a low-birth-weight infant (5). Studies from other parts of the world have shown that food transfers can improve energy intakes (6) and birth weight (mean difference + 73 g) when provided to pregnant women (7), whereas cash transfers tend to be more empowering for women and improve dietary diversity (3, 8–10).

However, it is possible that food and cash transfers would have differential impacts in South Asia than in other regions. Discrimination against women in the allocation of food is more pervasive in this region than elsewhere (11, 12), particularly during pregnancy (13), and this may prevent women from consuming transfers delivered at the household level (14). Therefore, trials from other locations may not be valid for South Asia, and interventions may need to incorporate additional components to change intrahousehold allocation practices (14).

Common approaches to change these food-related behaviors include nutrition education, counseling, and women's group interventions (15–17). One potential intervention is women's groups using a “Participatory Learning and Action” (PLA) approach (18), in which group members discuss and prioritize health issues, identify local barriers to good health, and implement and informally evaluate strategies to address these barriers (16, 19). A meta-analysis found that PLA women's groups reduced neonatal mortality by 20%, and by 33% when >30% of the group members were pregnant (20), but impacts on nutritional outcomes are weaker (21, 22). To better improve nutritional outcomes, women's group members may require additional resources, such as food or cash transfers, so they can act upon new knowledge and skills.

The Low Birth Weight South Asia Trial (LBWSAT) tested the effects of monthly PLA women's groups, with or without cash [750 Nepalese rupees (NPR); US$7.5/mo] or food (10 kg fortified wheat-soya “Super Cereal”/mo) transfers during pregnancy in Nepal, on birth weight (≤72 h after birth) and weight-for-age *z* scores (children aged 0–16 mo) (23). Birth weight improved by 78 g (95% CI: 13.9, 142 g) in the PLA plus food arm but did not significantly improve in the PLA-only or PLA plus cash arms (24), nor were there any effects on weight-for-age in any arms. This substudy, nested within LBWSAT, aimed to understand how these interventions influenced dietary patterns and whether they selectively targeted pregnant women, by measuring the effects on maternal dietary adequacy, intrahousehold food allocation, and nutritional status during pregnancy.

## Methods

### Study setting and population

The trial (ISRCTN 75964374) was implemented in the Dhanusha and Mahottari districts (in province 2) in Nepal by University College London and a Nepalese research organization, Mother and Infant Research Activities (MIRA), in collaboration with the World Food Programme and Save the Children UK. These districts were purposively sampled because the burden of undernutrition is high: 28% of infants are born with a low birth weight (25) and 29% of women are underweight (26).

Dhanusha and Mahottari districts have a combined population of ∼1.4 million (27). Flooding is frequent during the monsoon, and poor-quality roads, high temperatures, and humidity make travel to study clusters difficult. The study period coincided with the aftermath of the 2015 earthquakes and severe political unrest associated with Nepal's new constitution (28). This caused strikes, violent protests, road blockages, closure of markets and banks, and personal insecurity for the field team, making data collection difficult.

### Random allocation

The interventions were community based, so were allocated by cluster. Each cluster was defined as 1 Village Development Committee, a geopolitical unit that contains 9 wards. Clusters were excluded if they had been allocated to a women's group intervention in an earlier trial. To reduce the heterogeneity of the sample, clusters were also excluded if they were small (population <4000), large (population >9200), near to the East-West Highway, hilly, forested, non–Maithili-speaking, or large towns or municipalities. In a public “bingo” event involving local stakeholders, the remaining 80 clusters were allocated to 1 of 4 trial arms, giving 20 clusters per arm. We used stratified block randomization. Strata were based on cluster size and accessibility: small inaccessible, small accessible, large inaccessible, and large accessible. Due to the nature of the interventions, masking of the cluster allocations was not possible. Statisticians were not masked to the allocation; analyses followed a prespecified plan.

### Participants

All pregnant women living in selected clusters were eligible to participate in the trial, but this study evaluated the effects of the interventions on diets and intrahousehold food allocation in joint, male-headed households. This was to reduce the heterogeneity of the sample and because these households were hypothesized to be the least equitable and least likely to change their behaviors (14). Criteria for enrolled women and their households to be interviewed for this study were as follows: women in their third trimester who were living with their mother-in-law in male-headed households. Sampled individuals from these households were pregnant women, their mothers-in-law, and male household heads. Male household heads were hypothesized to be the most favored in terms of food allocation, and mothers-in-law typically controlled food allocation (13). We initially planned to collect data over 1 y but were delayed due to severe political conflict, major earthquakes, and lack of funds.

### Interventions

The full protocol is reported in Saville et al. (23). The 3 interventions were a behavior-change strategy of monthly women's groups practicing PLA, PLA women's groups with a monthly unconditional food transfer, and PLA women's groups with a monthly unconditional cash transfer.

**FIGURE 1:**
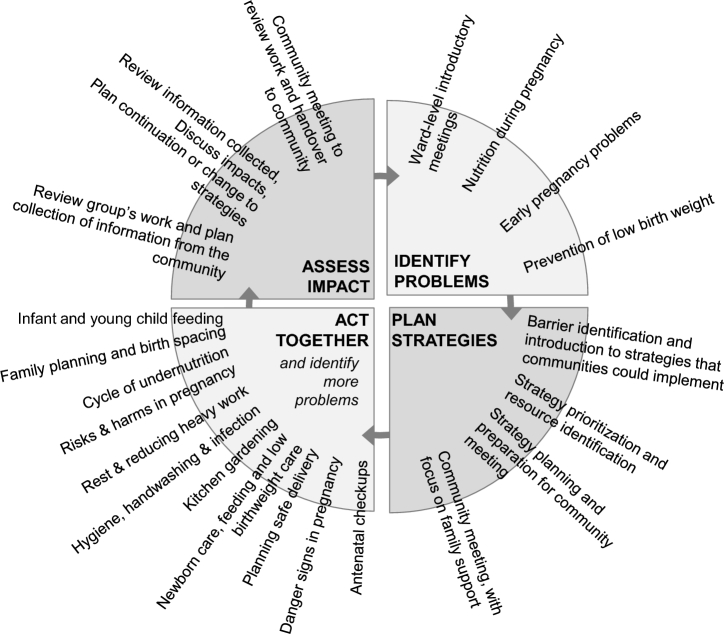
Women's group meeting plan using a Participatory Learning and Action cycle.

The groups followed a PLA cycle of 4 phases: identify problems, plan strategies, act together, and evaluate impact (as shown in **Figure 1**).

 In the first phase, groups used participatory methods such as picture cards, games, and stories to discuss nutrition problems and local barriers to achieving good health during pregnancy. In the second phase, groups prioritized and voted on the issues they wanted to focus on, designed strategies to address these problems, and engaged the wider local community for support and feedback. In the third phase, the groups implemented these strategies while continually discussing new topics related to pregnancy and infant health. Finally, in the fourth phase, the groups reviewed what went well and discussed what to do next after the implementing organization (MIRA) withdrew from the community.

The women's groups were held at the ward level and, with 9 wards per Village Development Committee, 539 women's groups were facilitated in all 60 intervention clusters (one small ward was merged with a neighboring ward). Government-incentivized female community health volunteers (FCHVs) facilitated the PLA women's groups, with support from MIRA-recruited “nutrition mobilizers.” FCHVs and nutrition mobilizers received training on group facilitation and maternal and infant nutrition.

The PLA groups served as a mechanism to deliver the food and cash transfers. The food consisted of 10 kg of fortified wheat and soya blend with sugar (“Super Cereal”)—a product developed and tested by the World Food Programme—and the cash was NPR 750 (23). The nutritional composition and safety of Super Cereal is reported in the trial protocol (23). Women were advised to eat 150 g Super Cereal/d, which would provide 570 kcal/d and meet most micronutrient requirements for pregnant women. The monthly 10-kg transfer allowed for women to share another 179 g/d with other household members. The cash transfer was calculated to be the equivalent cost of the Super Cereal transfer. The cost of an adequate diet in 2012 was estimated at NPR 1767/mo per person, so the cash transfer would provide 43% of this cost. In the PLA plus food arm, PLA groups discussed how much Super Cereal should be consumed (150 g/d) and recipes for using the Super Cereal. In the PLA plus cash arm, groups discussed how the cash should be spent on nutritious foods such as milk and fruit. In both transfer arms, groups discussed why the transfers should be selectively consumed by pregnant women.

As a benefit to all participants (including controls), training on maternal, newborn, and child health was provided to 189 health workers in both districts. When the final measurements were taken after delivery, women in the control and PLA-only arms received NPR 1000 to thank them for participating.

### Outcomes

The outcomes are summarized in **Table 1**.

**TABLE 1:** Primary and secondary outcomes^1^

Outcome	Measure
Primary outcomes	• RDEAR between pregnant women and male household heads
	• RDEAR between pregnant women and mothers-in-law
Secondary outcomes on intrahousehold food allocation	
Nutrient allocation	• Relative dietary iron adequacy ratio between pregnant women and household heads
	• Relative dietary iron adequacy ratio between pregnant women and mothers-in-law
	• Relative total iron adequacy ratio between pregnant women and household heads
	• Relative total iron adequacy ratio between pregnant women and mothers-in-law
	• Ratio of MPA between pregnant women and household heads^2^
	• Ratio of MPA between pregnant women and mothers-in-law^2^
Food item allocation	• Odds of pregnant women consuming more flesh foods than household heads^3^,^4^
	• Odds of pregnant women consuming more flesh foods than mothers-in-law^3^,^4^
	• Odds of pregnant women consuming more dairy foods than household heads^4^
	• Odds of pregnant women consuming more dairy foods than mothers-in-law^4^
	• Odds of pregnant women consuming more GLVs than household heads^4^
	• Odds of pregnant women consuming more GLVs than mothers-in-law^4^
Secondary outcomes for pregnant women	
Food items	• Odds of pregnant women consuming any flesh foods^3^
	• Odds of pregnant women consuming any dairy foods
	• Odds of pregnant women consuming any GLVs
	• Odds of consuming any iron-folate supplements
	• MDD-W over 1-d recall period^2^,^5^
Nutrients	• Kilocalorie intake
	• Kilocalorie adequacy ratio (intakes/Estimated Average Requirements)
	• Protein adequacy ratio (intakes/Estimated Average Requirements)
	• Dietary iron adequacy ratio, without supplements (intakes/recommended intakes)
	• MPA, averaged across 11 micronutrients^2^
Nutritional status	• MUAC, cm

^1^GLV, green leafy vegetable; MDD-W, Minimum Dietary Diversity of Women; MPA, mean probability of adequacy for 11 micronutrients; MUAC, mid-upper arm circumference; RDEAR, Relative Dietary Energy Adequacy Ratio.

^2^Added to initial analysis plan at a later stage.

^3^Flesh foods include any type of meat, fish, or shellfish (not eggs).

^4^Binary categories of pregnant women consuming more, compared with the same or less than the compared household member, based on the average over the 3-d recall period.

^5^MDD-W was based on the first day of dietary recall (rather than all 3 recall days) to use a comparable recall period for which this score was validated.

We report intervention effects on food and nutrient intakes of pregnant women and intrahousehold allocation. To describe both intakes and allocation, we selected nutrients (energy, protein, and dietary iron) and food groups (“flesh foods”, i.e., meat, fish, or shellfish; dairy foods; and green leafy vegetables) that were promoted in the PLA groups. Other foods were also promoted, but we chose flesh foods because they are culturally high status, dairy foods because they are high-status foods that are cheaper and more regularly consumed than flesh foods, and green leafy vegetables because they are micronutrient-rich yet considered “inferior.” We report on intakes of dietary iron (excluding iron-folate supplements) and consumption of supplements, and on intrahousehold allocation of dietary and total iron (dietary iron plus iron from supplements). Midupper arm circumference (MUAC) is reported as an indicator of nutritional status.

We also report summary measures that were added to the initial analysis plan to describe overall nutritional and dietary adequacy: mean probability of adequacy (MPA), the average adequacy of 11 nutrients, and the Minimum Dietary Diversity Score for Women (29), the number of food groups consumed out of 10 food groups.

The primary outcomes for this study were mean Relative Dietary Energy Adequacy Ratio (RDEAR) between pregnant women and the male household heads and mean RDEARs between pregnant women and their mothers-in-law, as defined in the following equation:

(1) \begin{eqnarray*} RDEAR\ &=& \left( {\frac{{kcal\ intake{\ _a}}}{{kcal\ Estimated\ Average\ Requirements{\ _a}}}} \right)/\nonumber\\ &&\left( {\frac{{kcal\ intake{\ _b}}}{{kcal\ Estimated\ Average\ Requirements{\ _b}}}} \right); \end{eqnarray*}

where *a* denotes the pregnant woman and *b* denotes another household member (the male household head or mother-in-law). Estimated Average Requirements (EARs) were individually calculated, on the basis of their age, sex, body weight, pregnancy/lactating status, and self-reported physical activity levels (30). Total energy expenditure was calculated as basal metabolic rate × physical activity levels. Basal metabolic rates were based on Indian-specific values (31). Self-reported activity levels were assessed by asking each respondent whether they spent most of the previous day *1*) sitting down or standing still, such as doing office or shop work (“sedentary”); *2*) moving around, such as walking, carrying light loads, or doing domestic work (“moderate”); or *3*) doing strenuous work like carrying heavy loads, working in the field, or pulling a rickshaw (“strenuous”). Based on Indian Council of Medical Research's guidelines, Physical activity levels were taken as 1.5 for sedentary activity, 1.8 for moderate work, and 2.3 for strenuous, heavy activity levels (31). The additional cost of pregnancy was taken to be 390 kcal/d (31).

We calculated the ratios of the MPA between pairs of household members. MPA was calculated by using the Probability approach (32), as follows. We first transformed nutrient intake distributions to normality using Box-Cox transformations (33) and obtained the best linear unbiased predictors derived from separate linear mixed-effects regression models for each household member, treating clusters and individuals (repeat measurements) as random effects and strata and study arm as fixed effects. We then calculated the probability of adequacy (PA) for each individual and each nutrient by comparing the individual's back-transformed usual intake to the population distribution of requirements, which are normal distributions with known means (i.e., EARs) and SDs (34–37). For mothers-in-law and male household heads, iron PAs were calculated by using a table of probabilities for different intervals of usual intakes (36). We assumed low bioavailability of iron (5%), apart from pregnant women who have higher iron absorption (23%) (36), and low bioavailability of zinc (25% for women, 18% for men) (37).

We also report, but did not formally test, the percentage of women with a probability of iron adequacy of >0.7 based on total iron including supplements.

### Field procedures

Enrollment of pregnant women into the main trial ran from December 2013 to February 2015, and the interventions ran from February 2014 to October 2015. The last enrolled woman gave birth in October 2015. Between 10 June and 26 September 2015, all eligible households were visited for a series of three 24-h dietary recall interviews. Dietary intakes of pregnant women, male household heads, and mothers-in-law were assessed up to 3 times each on nonconsecutive days. The 5-stage “multi-pass” dietary assessment method has been described in detail elsewhere (38–40). Portion sizes were estimated by using an atlas of life-sized photographs that was developed locally and validated before use (41).

Individual nutritional intakes were estimated with the use of a local food-composition table compiled from multiple published sources (42–45). We also added the nutritional content of cooked, mixed dishes to this table using recipes that we collected before data collection and analyzed using the published values of raw foods. We did not apply nutrient retention factors due to the lack of locally appropriate estimates.

MUAC and body weight were measured with the use of Seca 212 circumference tapes and Tanita solar weighing scales accurate to 100 g. Socioeconomic and demographic data were collected by the main trial surveillance (23, 46). Data on exposure to the interventions were collected during the third dietary recall.

Before data collection, interviewers received standardization exercises for MUAC measurement and 1 wk of training on dietary assessment. To ensure data quality, supervisors and project coordinators conducted spot checks, revisited interviewed households, and checked plausibility of values, frequency of outliers, digit preference, and Global Positioning System points every week.

### Ethics

Research ethics approval was obtained from the Nepal Health Research Council (108/2012) and the University College London Ethical Review Committee (4198/001). Women gave consent by signature or thumbprint. A data-monitoring committee was formed, and harms were tracked in the form of stillbirths and deaths, but there was no intent to ask the committee to apply any stopping rules, and no reason to believe the interventions would cause harm (23). For each 24-h recall interview, respondents gave verbal consent and were free to stop the interviews at any point.

### Statistical methods

#### Sample size

The target sample size was 200 households per arm, to detect a difference in the primary outcome (RDEAR) of 0.1 from 0.9 to 1.0 between 2 trial arms, with 80% power and 95% confidence. This assumed 19 clusters (allowing loss of 1 cluster) per arm, an SD of 0.27, an intracluster correlation coefficient of 0.03, and a 70% response rate.

#### Main analysis methods

Main analyses were performed under the intention-to-treat principle, so included all sampled households irrespective of their exposure to the interventions. Effect sizes (regression coefficient or OR) were estimated by fitting linear or logistic mixed-effects regression models. We treated clusters as a random effect and strata as a fixed effect. Allocation ratios were log-transformed to give normally distributed residuals. For log-transformed outcomes, effect sizes of transformed outcomes are reported in the tables, but the exponents are provided in the text for interpretability. Effect sizes, 95% CIs, and *P* values are reported. Cluster-adjusted chi-square tests assessed differences in characteristics between respondents and nonrespondents.

We included outliers (>3500 or <1000 kcal) in energy intakes and data from respondents who were fasting or feasting; results were very similar when outliers were excluded. For MPA ratios only, we had some extreme outliers in log-transformed MPA ratios (values <−8), which we excluded to give normally distributed residuals.

#### Covariate adjustment

To select covariate adjustments, goodness-of-fit for nested models was assessed with the use of Wald tests, comparing models with and without the covariate and excluding covariates in the model using a backward-stepwise method. Covariates included in the models were as follows: caste-religion group (3 categories), tertiles of a wealth score (the first component from a principal components analysis based on ownership of assets), maternal education, husband living overseas, and a binary variable indicating whether the interview was conducted before or during the monsoon season. Log-RDEAR was also included as a covariate in analyses for iron, MPA, and food item allocation. The following covariates were tested but excluded due to a lack of significant association with the outcomes: maternal age, gestational age, and household size. Adjusted analyses had a lower number of observations; 16 of 805 households were dropped due to missing data on overseas migration.

#### Dose-response

A dose-response analysis tested the effect of high exposure compared with lower exposure for outcomes from those primary analyses (RDEARs) showing significant effects. We compared the effect of pregnant women receiving ≥6 transfers with <6 transfers and any household member attending the group at least once with no one ever attending the groups.

The significance level for all hypothesis testing was set at 0.05, with no formal adjustment for multiple comparisons. All of the analyses were conducted with Stata SE 14 (StataCorp LP).

## Results

### Response rate and respondent characteristics

**FIGURE 2:**
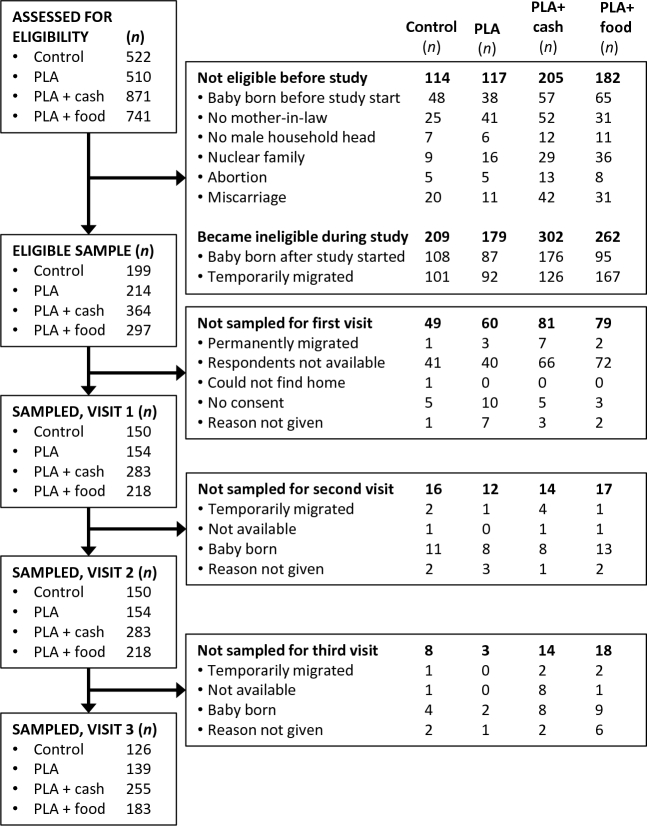
Reasons for nonresponse by trial arm. PLA, Participatory Learning and Action.

Reasons for nonresponse are shown in **Figure 2**.

 One cluster in the PLA-only arm had no data because of the poor performance of an enumerator who was responsible for enrolling women in that cluster. Of the 2644 trial participants assessed for eligibility, 618 (23%) were not eligible. Reasons for this included the following: infant being born before the dietary recall survey began, no mother-in-law in the household, no male household head, pregnant woman living in a nuclear family, or pregnant woman miscarried or had an abortion.

Some women (*n *= 952) became ineligible during the survey period and so were not sampled, either because they were temporarily not living with their mother-in-law or because they gave birth. It is a common tradition for women to move to their natal home for support during the last few weeks of pregnancy and birth and postpartum periods. The study period also coincided with an infrequent, month-long tradition, *malamas*, during which women avoid traveling and tend to stay at their natal home. Other women were eligible but not sampled (*n *= 269), because they could not be found, declined to participate, permanently migrated, or were not available.

**Table 2** shows characteristics of respondents and nonrespondents.

**TABLE 2:** Characteristics of respondents and nonrespondents^1^

		Respondents vs. nonrespondents,^2^ %			Trial arm,^3^ %			
	Total eligible at start of study, %	Became ineligible during study	Eligible but not sampled	Sampled in all arms	Control	PLA	PLA + cash	PLA + food
*n*	2026	952	269	805	150	154	283	218
Age of pregnant woman (*n *= 2015)^4^								
<19 y	11.5	12.9	7.5	11.2	12.7	14.9	10.6	8.3
19–29 y	81.9	79.1	82.7	84.8	84.0	83.1	83.8	88.1
>29 y	6.6	7.9	9.8	4.0	3.3	2.0	5.7	3.7
Gravidity (*n *= 1945)^5^								
Primigravid	35.8	38.4	29.6	35.0	34.5	31.9	33.8	29.2
Multigravid	64.2	61.6	70.4	65.0	65.5	68.1	66.2	70.8
Caste group (*n* = 2015)^6^								
Dalit and Muslim (disadvantaged groups)	34.9	37.7	38.3	30.4	35.3	32.5	29.3	27.1
*Janjati*/other *Terai* castes	42.2	40.1	42.1	44.7	42.7	54.5	41.0	44.0
High caste (*Yadav, Brahmin*)	22.9	22.1	19.5	24.8	22.0	13.0	29.7	28.9
Principal component wealth score of 12 items (*n *= 1981)^7^								
Lowest tertile	31.5	33.0	38.1	27.8	36.5	39.9	27.4	34.4
Middle tertile	33.1	31.8	30.4	35.5	33.1	30.1	35.9	32.6
Highest tertile	35.3	35.2	31.5	36.8	30.4	30.1	36.7	33.0
Land ownership (*n *= 1981)^8^								
Landless	31.2	31.5	39.6	28.0	34.5	34.6	21.7	27.1
Owns land	68.9	68.5	60.4	72.0	65.5	65.4	78.3	72.9
Maternal education (*n *= 1982)^9^								
Never went to school	58.8	61.6	63.5	54.0	56.1	55.6	52.3	53.7
Primary to lower secondary	22.8	20.7	22.7	25.2	27.0	26.1	27.4	20.6
Secondary and above	18.4	17.7	13.8	20.8	16.9	18.3	20.3	25.7
Husband's education (*n *= 1981)^10^								
Never went to school	41.9	44.7	46.3	37.4	42.2	34.4	39.6	30.7
Primary to lower secondary	32.3	30.9	32.8	33.6	29.9	34.4	33.9	36.7
Secondary and above	25.8	24.4	20.8	29.0	27.9	31.1	26.4	32.6
Overseas migration (*n *= 1941)^11^								
Husband not living overseas	80.9	78.7	81.6	83.3	78.8	81.3	87.7	81.9
Husband living overseas	19.1	21.3	18.4	16.7	21.2	18.7	12.3	18.1

^1^PLA, Participatory Learning and Action.

^2^Sampled respondents represent the reference category, in cluster-adjusted chi-square tests for differences between respondents and nonrespondents.

^3^Response rates for characteristics of sampled households ranged between 98% (789 of 805 for overseas migration) and 100%.

^4^Cluster-adjusted χ^2^ test comparing respondents and non-respondents = 8.80; *P *= 0.066.

^5^Cluster-adjusted χ^2^ test comparing respondents and non-respondents = 4.47; *P *= 0.107.

^6^Cluster-adjusted χ^2^ test comparing respondents and non-respondents = 3.14; *P *= 0.534.

^7^Cluster-adjusted χ^2^ test comparing respondents and non-respondents = 3.19; *P *= 0.527.

^8^Cluster-adjusted χ^2^ test comparing respondents and non-respondents = 4.35; *P *= 0.114.

^9^Cluster-adjusted χ^2^ test comparing respondents and non-respondents = 7.71; *P *= 0.103.

^10^Cluster-adjusted χ^2^ test comparing respondents and non-respondents = 5.39; *P *= 0.250.

^11^Cluster-adjusted χ^2^ test comparing respondents and non-respondents = 2.35; *P *= 0.310.

There were no significant differences between sampled and nonsampled respondents. The average ages of pregnant women, household heads, and mothers-in-law were 22, 43, and 50 y, respectively. Approximately one-third (30%) were Dalit or Muslim households, 45% were *Janjati* (indigenous) or other plains ethnicity castes, and the rest were high caste. More than half (54%) of pregnant women had no schooling, 37% of their husbands had no schooling, and almost one-third (28%) were landless.

### Exposure to interventions

Group attendance was much higher in the transfer arms (86% and 96% attendance in the food and cash arms, respectively) than in the PLA-only arm (25%) (**Table 3**). Groups were most commonly attended by pregnant women and their mothers-in-law. Most women in the transfer arms had received ≥4 transfers of either cash or food by their third dietary recall.

**TABLE 3:** Exposure to PLA groups by arm, and receipt of food and cash transfers^1^

	Trial arm, %			
	Control	PLA	PLA+cash	PLA+food
Ever attended women's groups by any family member	1.6	24.7	96.1	86.0
Pregnant woman ever attended	1.6	17.8	95.7	85.0
Mother-in-law ever attended	0	13.0	72.8	67.3
Husband ever attended	0	0	0.7	1.4
Other family member ever attended	0	3.4	22.9	14.5
Transfer exposure				
0–3 transfers			2.5	7.4
4–5 transfers			37.9	60.9
6–7 transfers			59.6	31.7

^1^Response rates were 99% for women's groups, 98% for cash transfers, and 93% for food transfers. PLA, Participatory Learning and Action.

In the food arm, more than half (54%) of pregnant women consumed some Super Cereal, but only 3% consumed the recommended 150 g. Of those who consumed any, the median intake was 43 g. Most (55%) of the households used their Super Cereal to prepare *haluwa*, a stiff porridge prepared with oil and sugar (215 kcal/100 g); one-third (35%) prepared *roti*, unleavened flatbread (263 kcal/100 g); and 5% prepared *litho*, a watery Super Cereal mixture (82 kcal/100 g; estimates of nutritional values are based on typical average recipes). The median Super Cereal consumption of 43 g would give 165 kcal (or 42% of 390 kcal, the energy costs of pregnancy). Pregnant women in the PLA plus food arm consumed 2147 kcal/d; to meet EARs, pregnant women would need to consume an additional 256 kcal, which is 119 g *haluwa*, 97 g *roti* (around 2 small *roti*), or 312 g *litho* (around 1 medium-sized bowl). Six percent of household heads consumed some Super Cereal, as did 19% of mothers-in-law.

### Trial effects on dietary intakes of pregnant women

**Table 4** shows effects of the interventions on pregnant women's intakes of key nutrients and nutritional status (MUAC), and **Table 5** shows effects on food item consumption. **Supplemental Table 1** reports arm-wise intakes of energy, protein, and 11 micronutrients, MPA, dietary diversity, and MUAC. **Supplemental Table 2** reports the percentage of pregnant women consuming any of the 10 food groups or iron-folate supplements in each arm.

**TABLE 4:** Effects of PLA groups, PLA with cash, and PLA with food, on nutrient intakes of pregnant women^1^

		PLA		PLA+cash		PLA+food	
Outcome	Control^2^	Adjusted difference relative to control (95% CI)	*P*	Adjusted difference relative to control (95% CI)	*P*	Adjusted difference relative to control (95% CI)	*P*
Kilocalories/d^3^	2239 ± 730 (2146)	−118 (−329, 94)	0.275	−80 (−276, 117)	0.427	86 (−114, 286)	0.399
Kilocalorie adequacy ratio/d^3^	0.88 ± 0.29 (0.84)	−0.07 (−0.15, 0.01)	0.090	−0.05 (−0.12, 0.03)	0.242	0.04 (−0.03, 0.12)	0.279
Protein adequacy ratio/d^4^	1.32 ± 0.46 (1.30)	0.01 (−0.02, 0.03)	0.577	0.03 (−0.00, 0.06)	0.069	−0.00 (−0.03, 0.03)	0.929
Dietary iron adequacy ratio/d^4^	0.42 ± 0.15 (0.42)	0.01 (−0.05, 0.07)	0.723	0.06 (0.01, 0.11)	0.015	0.07 (0.01, 0.12)	0.012
MPA^5^	0.37 ± 0.20 (0.36)	0.05 (0.00, 0.09)	0.044	0.04 (0.00, 0.09)	0.037	−0.01 (−0.05, 0.03)	0.639
MDD-W (score of 0 to 10)^5^	4.6 ± 1.2 (5.0)	0.05 (−0.25, 0.36)	0.725	0.35 (0.08, 0.63)	0.012	0.14 (−0.14, 0.43)	0.316
MUAC,^3^ cm	23.5 ± 2.1 (23.5)	0.75 (0.28, 1.23)	0.002	0.75 (0.33, 1.17)	<0.001	0.49 (0.05, 0.93)	0.029

^1^Values for the control arm are means±SDs (medians), and effect sizes are adjusted differences (95% CIs) relative to control. MDD-W, Minimum Dietary Diversity for Women; MPA, mean probability of adequacy; MUAC, midupper arm circumference; PLA, Participatory Learning and Action.

^2^Means ± SDs (medians) were calculated by using mean intakes of the 3 recalls, rather than “usual” intakes calculated from best linear unbiased predictors. *n* = 805.

^3^Multivariable linear regressions adjusted for clustering, strata, caste/religion, tertiles of a wealth score, maternal education, husband migrating overseas, monsoon season (vs, premonsoon). *n* = 789.

^4^Modeled with log-transformed outcome. Multivariable linear regressions adjusted for clustering, strata, caste/religion, tertiles of a wealth score, maternal education, husband migrating overseas, monsoon season (vs. premonsoon), and log-transformed kilocalorie adequacy ratio. *n* = 789.

^5^Multivariable linear regressions adjusted for clustering, strata, caste/religion, tertiles of a wealth score, maternal education, husband migrating overseas, monsoon season (vs. premonsoon), and kilocalorie adequacy ratio. *n* = 789.

**TABLE 5:** Effects of PLA groups, PLA with cash, and PLA with food on pregnant women's intakes of key food types and iron-folate supplements^1^

		Group^3^					
		PLA		PLA + cash		PLA + food	
Outcome	Control^2^	OR relative to control (95% CI)	*P*	OR relative to control (95% CI)	*P*	OR relative to control (95% CI)	*P*
Iron-folate supplements	28.6	2.53 (1.14,5.60)	*P* = 0.022	4.62 (2.19,9.78)	*P* < 0.001	3.08 (1.45,6.54)	*P* = 0.004
Flesh foods	32.7	1.28 (0.78,2.11)	*P* = 0.332	1.53 (0.98,2.40)	*P* = 0.062	1.39 (0.87,2.22)	*P* = 0.165
Dairy	68.0	0.65 (0.39,1.10)	*P* = 0.108	1.80 (1.09,2.98)	*P* = 0.022	0.79 (0.48,1.30)	*P* = 0.353
Green leafy vegetables	66.7	1.01 (0.55,1.86)	*P* = 0.974	1.11 (0.64,1.94)	*P* = 0.713	1.22 (0.68,2.17)	*P* = 0.502

^1^PLA, Participatory Learning and Action.

^2^Values for control arm are percentages, calculated based on any consumption over the repeated dietary recalls. *n* = 805.

^3^Effect sizes are adjusted ORs relative to control (95% CIs). Odds are based on any consumption over the repeated dietary recalls. Multivariable logit regression models adjusted for clustering, strata, caste/religion, tertiles of a wealth score, maternal education, husband migrating overseas, monsoon season (vs. premonsoon). *n* = 789.

Compared with the control, participants consumed, on average, 86 kcal more in the PLA plus food arm and 118 kcal less in the PLA-only arm, but neither these nor the slight differences in kilocalorie adequacy ratios were significant. Dietary iron adequacy was higher in both transfer interventions than in the control arm (PLA plus cash = coefficient of 0.06; 95% CI: 0.01, 0.11; *P* = 0.015; PLA plus food: 0.07; 95% CI: 0.01, 0.12; *P* = 0.012). Notably, in the PLA plus cash arm, odds of consuming iron-folate supplements were 4.6 times higher, the odds of consuming dairy were 1.8 times higher, and the mean dietary diversity and PA were higher than in the control arm. For all interventions, the pregnant women's mean MUAC and the odds of consuming iron-folate supplements were significantly higher than in the control arm.

### Trial effects on intrahousehold food allocation

The trial effects on intrahousehold nutrient allocation are given in **Table 6** (with the nutrient allocation ratios in each arm shown in **Supplemental Table 3**), and effects on intrahousehold food item allocation are reported in **Table 7** (with food allocation ratios in each arm given in **Supplemental Table 4**). Effect sizes are reported as log-transformed outcomes in the tables, but their exponents are reported in the text for ease of interpretation.

**TABLE 6:** Effects of PLA groups, PLA with cash, and PLA with food on intrahousehold nutrient allocation^1^

	Control			PLA		PLA + cash		PLA + food	
Outcome	*n*	Mean ± SD (median); normal scale	*n* in adjusted regression models	Adjusted difference relative to control (95% CI)	*P*	Adjusted difference relative to control (95% CI)	*P*	Adjusted difference relative to control (95% CI)	*P*
Pregnant women vs. household heads									
RDEAR^2^	803	0.86 ± 0.30 (0.83)	787	−0.11 (−0.19, −0.02)	0.020	−0.05 (−0.13, 0.03)	0.255	0.07 (−0.01, 0.16)	0.078
Relative dietary iron adequacy ratio^3^	803	0.40 ± 0.18 (0.37)	787	−0.01 (−0.07, 0.06)	0.821	0.04 (−0.02, 0.09)	0.195	0.04 (−0.02, 0.10)	0.157
Relative total iron adequacy ratio^3^	803	0.70 ± 0.82 (0.42)	787	0.21 (0.02, 0.41)	0.032	0.41 (0.23, 0.59)	<0.001	0.21 (0.03, 0.40)	0.022
MPA ratio^3^	801	0.68 ± 0.41 (0.62)	785	0.23 (0.01, 0.45)	0.038	0.16 (−0.04, 0.36)	0.123	−0.01 (−0.21, 0.20)	0.937
Pregnant women vs. mothers-in-law									
RDEAR^2^	799	0.91 ± 0.31 (0.88)	783	−0.07 (−0.16, 0.03)	0.183	0.04 (−0.05, 0.13)	0.336	0.12 (0.02, 0.21)	0.014
Relative dietary iron adequacy ratio^3^	800	0.63 ± 0.20 (0.60)	782	0.03 (−0.03, 0.09)	0.345	0.04 (−0.01, 0.09)	0.109	0.02 (−0.03, 0.08)	0.426
Relative total iron adequacy ratio^3^	800	1.08 ± 1.11 (0.65)	782	0.24 (0.03, 0.45)	0.027	0.41 (0.21, 0.60)	<0.001	0.18 (−0.02, 0.38)	0.078
MPA ratio^3^	802	0.73 ± 0.36 (0.68)	781	0.17(−0.05, 0.38)	0.136	−0.02 (−0.23, 0.18)	0.814	−0.10 (−0.30, 0.11)	0.366

^1^Values for the control arm are means on a normal scale ± SDs (medians), and effect sizes are adjusted differences of log-transformed outcomes relative to control (95% CIs). MPA, mean probability of adequacy; PLA, Participatory Learning and Action; RDEAR, Relative Dietary Energy Adequacy Ratio.

^2^Multivariable linear regression models adjusted for clustering, strata, caste/religion, tertiles of a wealth score, maternal education, husband migrating overseas, monsoon season (vs. premonsoon).

^3^Multivariable linear regression models adjusted for clustering, strata, caste/religion, tertiles of a wealth score, maternal education, husband migrating overseas, monsoon season (vs. premonsoon), and log-RDEAR.

**TABLE 7:** Effects of PLA groups, PLA with cash, and PLA with food on intrahousehold food allocation^1^

		PLA^3^		PLA + cash^3^		PLA + food^3^	
Outcome	Control^2^ % households where pregnant women ate more	OR relative to control (95% CI)	*P*	OR relative to control (95% CI)	*P*	OR relative to control (95% CI)	*P*
Pregnant women vs. household heads							
Flesh foods	7.3	2.36 (1.09, 5.10)	0.028	1.86 (0.90, 3.85)	0.093	2.20 (1.06, 4.55)	0.034
Dairy foods	27.3	1.07 (0.60, 1.93)	0.810	1.71 (1.02, 2.85)	0.041	1.12 (0.66, 1.89)	0.684
Green leafy vegetables	32.0	1.05 (0.63, 1.75)	0.844	1.26 (0.81, 1.96)	0.304	1.00 (0.63, 1.59)	0.995
Pregnant women vs. mothers-in-law							
Flesh foods	14.7	1.47 (0.79, 2.73)	0.227	1.47 (0.84, 2.57)	0.174	1.47 (0.82, 2.62)	0.193
Dairy foods	40.7	1.06 (0.63, 1.80)	0.825	1.30 (0.82, 2.07)	0.270	0.93 (0.57, 1.50)	0.759
Green leafy vegetables	33.3	1.13 (0.68, 1.88)	0.633	1.11 (0.71, 1.73)	0.649	1.22 (0.77,1.95)	0.392

^1^PLA, Participatory Learning and Action; RDEAR, Relative Dietary Energy Adequacy Ratio.

^2^Values are percentages of households where pregnant women ate more, based on average consumption of ≤3 dietary recalls per person. *n* = 805.

^3^Effect sizes are adjusted ORs relative to control (95% CIs), based on average consumption of ≤3 dietary recalls per person. Multivariable logit regression models adjusted for clustering, strata, caste/religion, tertiles of a wealth score, maternal education, husband migrating overseas, monsoon season (vs. premonsoon), and log-RDEAR. *n* = 787.

In the PLA plus food arm, RDEARs between pregnant women and their mothers-in-law were significantly higher (12% higher; *P* = 0.014) than in the control arm, and RDEARs between pregnant women and their household heads were slightly but not significantly higher (8% higher; *P* = 0.078). On the contrary, in the PLA-only arm, RDEARs between pregnant women and the male household heads were 10% lower (*P* = 0.020), but MPA ratios (after adjusting for RDEARs) were 27% higher (*P* = 0.038) compared with the control arm. In the PLA plus cash arm, there was no difference in RDEARs or MPA ratios between pregnant women and household heads or their mothers-in-law relative to control.

For total iron (including supplements), compared with the control arm, all intervention arms had higher allocation ratios between pregnant women and male household heads [51% higher for PLA plus cash (*P* < 0.001); 24% higher for PLA only (*P* = 0.032) and PLA plus food (*P* = 0.022)]. A similar trend was observed between pregnant women and their mothers-in-law. There were no trial effects on intrahousehold dietary iron allocation.

In all of the intervention arms, the odds of pregnant women consuming more animal-source foods (flesh foods and dairy) than the household head were higher than in the control arm, with the largest effect in the PLA-only arm (flesh food OR: 2.4; 95% CI: 1.09, 5.10; *P* = 0.028). There were no differences between trial arms in the odds of pregnant women consuming more flesh foods, dairy, or green leafy vegetables compared with their mothers-in-law.

### Dose-response

There were no significant effects of different levels of exposure within arms on RDEARs. All coefficients were nonsignificant (all *P* values >0.3; results not shown).

## Discussion

PLA groups, with and without food and cash transfers, are broadly beneficial for maternal nutrition and equity of intrahousehold food allocation, in a context in which the allocation of foods and nutrients are found to be highly inequitable. All of the interventions increased the consumption of iron-folate supplements, anthropometric status, and the intrahousehold allocation of some animal-source foods. The greatest effects on the intrahousehold allocation of energy between pregnant women and their mothers-in-law were in the PLA plus food arm, and the food transfers were channeled to pregnant women. However, the consumption of the food transfers was less than one-third of that received, and pregnant women's average energy intakes did not significantly increase. In the PLA plus cash arm we observed the greatest beneficial effects on dietary diversity and the consumption and allocation of iron and dairy foods. Micronutrient adequacy also improved slightly, although energy intakes did not improve, nor did the allocation of energy between pregnant women and other household members. PLA groups without transfers had mostly positive but mixed effects on diets and dietary allocation, with notable improvements in MPA, MPA allocation, and the allocation of flesh foods. However, PLA group attendance was low in this arm at this point in the trial, and we also found, surprisingly, a slightly less equitable allocation of energy between pregnant women and male household heads. Given that energy deficiency is likely to be a bigger predictor of low birth weight than micronutrient deficiencies, the effects on RDEARs may explain why the PLA plus food intervention improved birth weight but the other interventions did not (24). Particularly in the PLA plus cash arm, the interventions may have resulted in other improvements in micronutrient status that were not measured.

Our results are consistent with an international review that found that cash transfers increase food expenditures, whereas food transfers increase energy consumption (10), perhaps because people spend their cash on more expensive (possibly more micronutrient-rich) but less energy-dense foods than the foods provided in transfers. The agreement of our results with this international review suggests that the reported South Asian norms that may restrict the allocation of food and cash transfers (food sharing, control over cash) are similar in other contexts or that PLA groups changed these norms to become similar to other contexts.

The effects of our interventions on iron intake and allocation may be explained by increased demand for iron-folate supplements or increased supply by FCHVs, perhaps due to PLA messages. Women may have also spent their transfers on iron-rich foods or supplements if they did not get supplements for free from the FCHVs. The effects on dairy consumption and allocation in the PLA plus cash arm were expected because the PLA groups encouraged women to spend their cash transfers on milk. In addition to being nutrient-rich and affordable, women could purchase milk from door-to-door sellers, thereby overcoming barriers that women face in leaving their homes. Fruit consumption also appears to be higher in the PLA plus cash arm, mirroring the PLA messaging to consume fruit. The small impact on overall dietary diversity, but larger improvements in consumption of certain foods, also mirrors findings from a cash transfer program in Bangladesh that found no impact on child dietary diversity but higher consumption of protein-rich foods (47) and a cash transfer program from Pakistan that found minimally higher food consumption but large increases in meat and fruit consumption (48).

In the PLA plus food arm, the positive effect on RDEARs is consistent with the evidence that food transfers increase energy consumption (10). Other household members rarely consumed the Super Cereal, indicating that it was not given preferentially to the typically favored household heads. Although the Super Cereal may have been given to people who were not interviewed, the observed channeling to pregnant women reflects the messaging in the PLA groups: that the Super Cereal should be treated like a “women's medicine” for pregnancy. Qualitative research indicates that the Super Cereal was also considered inferior than the traditionally favored rice (49), although the Super Cereal was used to make foods that are acceptable and commonly consumed in this context. Different distribution and consumption amounts may have been observed if rice transfers were provided, such as the food-for-assets interventions by the World Food Programme in the west and far-west of Nepal (50). Indeed, a comparison of rice and wheat transfers from Bangladesh found that rice was preferentially given to men but wheat was channeled to women (9).

Despite this channeling toward pregnant women, it is not clear that their consumption of Super Cereal caused these changes in RDEARs. Overall, Super Cereal consumption was low, perhaps because the sugar added makes the flour taste different and it is harder to cook with than unfortified flour. In addition, women were sampled in their third trimesters and they may have felt too full to consume this energy-dense food at that stage of pregnancy. Different consumption patterns may have been observed in earlier trimesters, seasons, or earlier in the intervention period. Super Cereal may also have substituted other foods. This is difficult to determine because the difference in energy intakes between arms was small, although the consumption of other food groups in the PLA plus food arm appears mostly similar or higher than the PLA-only arm. An alternative explanation for the positive effects on RDEARs is that, because RDEARs account for energy requirements, the interventions caused reductions in pregnant women's workloads and energy requirements, as this was encouraged in the group meetings. However, exploratory analyses found no effect of any interventions on self-reported activity levels.

We cannot disentangle the effects of PLA groups and transfers within arms. Group attendance was higher in these arms because the transfers were delivered at the groups and so incentivized attendance. In addition, PLA groups may have influenced the allocation of transfers, by enabling or instructing women to spend the cash on themselves or to selectively consume the Super Cereal rather than contributing the transfers to a shared household pot (49).

The PLA groups without transfers had some effects on nutrition, including higher MUAC (0.8 cm), allocation of flesh foods to pregnant women, and odds of consuming of supplements. There may also have been some increases in egg consumption in all arms, which is surprising because, although eggs were promoted in the PLA meetings, they are considered unpopular in this cultural setting. The impacts of PLA groups are consistent with studies from Bangladesh and India that found slight, positive effects of PLA groups on diet diversity (21, 22). However, there were lower RDEARs between pregnant women and household heads in the PLA-only arm relative to control, which is surprising because groups were hypothesized to encourage better care and diets. The higher anthropometric status, perhaps caused by PLA groups in earlier trimesters, may have increased women's energy requirements and made adequacy and equity harder to achieve compared with the control arm. Nevertheless, the possibility that households became less equitable and that women consumed less in the PLA-only arm cannot be ignored.

Some study limitations must be considered. The response rate and sampling frame limit the external validity of our results. However, formative research indicates that the sampled households, in which the women were living with their in-laws, were the strictest and most inequitable (14). Therefore, the effect sizes may be a conservative estimate if extrapolated to the whole population. We were unable to disentangle the effects of different household members attending the groups. The high attendance by mothers-in-law could explain some of these dietary improvements for pregnant women, because mothers-in-law often dictate the allocation of foods. Alternatively, attendance by mothers-in-law may have posed a barrier to pregnant women making new friends and speaking up in the group meetings—key processes hypothesized to influence dietary behaviour change. Testing effects across multiple outcomes increases the risk of spuriously reporting significant results for individual outcomes, but the outcomes are not independent and we have interpreted the results holistically by considering all outcomes together. We initially planned to collect data over 1 y, which would have provided impact estimates that are representative of different seasons. Instead, the evaluation took place over 4 mo at the end of the trial, and enthusiasm toward the interventions might have waned; only 25% of women ever attended PLA groups, compared with 55% in the main trial. Larger effects might have been detected earlier, particularly for green leafy vegetables that were in season earlier in the year (51). On the other hand, evaluation at this point allowed time for the interventions to have worked at individual, household, and community levels. We did not apply nutrient retention factors, which could bias estimates if the interventions selectively increased consumption of raw or cooked foods, although we expect this potential bias to be minimal. The use of average recipes prevents us from detecting effects on recipe composition and portions, such as channeling the choicest pieces of meat. Finally, we only sampled 3 household members; the interventions may have differentially influenced food allocation between pregnant women and junior members of the household.

In summary, all of our PLA interventions (with or without transfers) improved diets and MUAC for pregnant women. The PLA with cash improved dietary diversity and adequacy, whereas PLA with food improved equity in energy allocation. PLA interventions with and without cash or supplements have an important role to play in marginalized populations where food security and nutrition for pregnant women are poor. Scale-up is a policy option, but more research is needed to understand and improve participants’ perceptions of and adherence to the interventions.

## Supplementary Material

Supplemental File

## Notes

Supported by the Child Health Research Appeal Trust (CHRAT), and UK Department for International Development (DFID; grant PO 5675). Some work for this study was done at Great Ormond Street Hospital and the University College London Great Ormond Street Institute of Child Health, which received funding from the UK Department of Health's National Institute for Health Research (NIHR) Biomedical Research Centers funding scheme. Partial funding of co-authors (NMS and AMdLC) was provided by the Wellcome Trust Strategic Award for the Population Science of Maternal and Newborn Survival (grant 085417MA/Z/08/Z)

Author disclosures: HAH-F, PP, TH, NS, SJ, BJB, AC, BPS, DSM, AMdLC, MC-B, and NMS, no conflicts of interest. None of the funders had any role in the design, analysis, or writing of this article.

Supplemental Tables 1–4 are available from the “Supplementary data” link in the online posting of the article and from the same link in the online table of contents at https://academic.oup.com/jn/.

**Abbreviations used**
EAR: Estimated Average Requirement
FCHV: female community health volunteer
MIRA: Mother and Infant Research Activities
MPA: mean probability of adequacy
MUAC: midupper arm circumference
NPR: Nepalese rupees
PA: probability of adequacy
PLA: Participatory Learning and Action
RDEAR: Relative Dietary Energy Adequacy Ratio
