# Supplementary material for: Participatory Women's Groups with Cash Transfers Can Increase Dietary Diversity and Micronutrient Adequacy during Pregnancy, whereas Women's Groups with Food Transfers Can Increase Equity in Intrahousehold Energy Allocation
Source: J Nutr. 2018 Jul 20;148(9):1472–83. doi: 10.1093/jn/nxy109 (PMC6118166; doi:10.1093/jn/nxy109)
Supplement: Supplemental File [file nxy109_supplement_file.docx]

**Supplemental table 1: Nutrient intakes, nutrient adequacy, dietary diversity, and nutritional status of pregnant women in each study arm**

|  |  | **Mean ± SD (median)** | | | |
| --- | --- | --- | --- | --- | --- |
| **Outcome** | ***n*** | **Control** | **PLA** | **PLA + cash** | **PLA + food** |
| Kcal/d | 805 | 2239± 730 (2146) | 2102± 635 (2107) | 2147± 700 (2061) | 2302± 708 (2205) |
| Kcal adequacy ratio/d | 805 | 0.88± 0.29 (0.84) | 0.80± 0.24 (0.79) | 0.82± 0.26 (0.80) | 0.91± 0.29 (0.89) |
| Protein, g/d | 805 | 67.8± 24.2 (65.2) | 64.3± 21.5 (63.5) | 67.2± 22.2 (64.1) | 70.5± 21.7 (70.0) |
| Protein adequacy ratio/d | 805 | 1.32± 0.46 (1.30) | 1.22± 0.39 (1.22) | 1.28± 0.42 (1.22) | 1.36± 0.42 (1.33) |
| Dietary iron, mg/d | 805 | 14.8±5.2 (14.6) | 13.8±4.3 (13.7) | 15.2±5.9 (14.5) | 16.3±5.8 (16.0) |
| Dietary iron adequacy ratio/d | 805 | 0.42± 0.15 (0.42) | 0.39± 0.12 (0.39) | 0.43± 0.17 (0.41) | 0.47± 0.16 (0.46) |
| % Total iron (including supplements) PA >0.7 | 805 | 16% | 21% | 28% | 23% |
| Vitamin C, mg/d | 805 | 133± 144 (96) | 117± 85 (99) | 137±128 (102) | 154±113 (125) |
| Vitamin A RE/d | 805 | 486±449 (359) | 454±401 (362) | 498±502 (386) | 498±513 (366) |
| Thiamin, mg/d | 805 | 1.5±0.7 (1.5) | 1.4±0.5 (1.4) | 1.5±0.6 (1.5) | 1.6±1.0 (1.5) |
| Riboflavin, mg/d | 805 | 1.1±0.6 (1.0) | 1.0±0.5 (0.9) | 1.3±0.7 (1.1) | 1.6±1.0 (1.4) |
| Niacin, mg/d | 805 | 16.3± 7.1 (15.0) | 15.1± 5.3 (14.2) | 15.9± 6.2 (15.1) | 18.2± 9.4 (17.2) |
| Vitamin B_6_, mg/d | 805 | 2.2±0.8 (2.1) | 2.0±0.6 (2.0) | 2.1±0.8 (2.0) | 2.4±0.9 (2.2) |
| Folate, µg/d | 805 | 639± 624 (383) | 961± 1092 (507) | 1092± 963 (936) | 996± 853 (715) |
| Vitamin B_12_, µg/d | 805 | 0.8± 0.9 (0.4) | 0.7± 0.9 (0.4) | 1.1± 1.7 (0.7) | 1.6± 1.9 (1.2) |
| Zinc, mg/d | 805 | 11.3± 4.0 (10.9) | 11.5± 4.2 (10.7) | 12.1± 4.6 (11.3) | 13.6± 4.6 (13.1) |
| Calcium, mg/d | 805 | 654± 462 (505) | 606± 405 (474) | 768± 473 (645) | 843± 564 (710) |
| MPA | 805 | 0.37± 0.20 (0.36) | 0.38± 0.18 (0.39) | 0.40± 0.20 (0.41) | 0.39± 0.20 (0.38) |
| MDD-W  (score of 0 to 10) | 805 | 4.6± 1.2 (5.0) | 4.6± 1.2 (5.0) | 5.0± 1.2 (5.0) | 4.8± 1.2 (5.0) |
| MUAC, cm | 805 | 23.5± 2.1 (23.5) | 24.3± 2.1 (23.9) | 24.4± 2.1 (24.1) | 24.1± 2.0 (23.9) |
| Values are reported as mean ± SD (median), calculated using mean intakes of the three recalls, rather than ‘usual’ intakes calculated from best linear unbiased predictors.  PA= Probability of Adequacy; MPA= Mean Probability of Adequacy; MDD-W= Minimum Dietary Diversity for Women; MUAC= Mid-upper arm circumference; PLA= Participatory Learning and Action; | | | | | |

**Supplemental table 2: Consumption of food groups and iron-folate supplements by pregnant women in each study arm**

|  |  | **% consuming any** | | | |
| --- | --- | --- | --- | --- | --- |
| **Outcome** | ***n*** | **Control** | **PLA** | **PLA + cash** | **PLA + food** |
| Iron-folate supplements | 805 | 28.6 | 44.2 | 61.8 | 54.1 |
| Flesh foods | 805 | 32.7 | 40.9 | 39.2 | 37.6 |
| Dairy | 805 | 68.0 | 58.4 | 79.2 | 66.1 |
| Green leafy vegetables | 805 | 66.7 | 67.5 | 69.3 | 71.1 |
| Starchy foods | 805 | 100.0 | 100.0 | 100.0 | 100.0 |
| Pulses | 805 | 95.3 | 94.2 | 97.2 | 99.1 |
| Nuts and seeds | 805 | 31.3 | 27.3 | 35.3 | 29.8 |
| Eggs | 805 | 18.0 | 26.6 | 25.1 | 21.6 |
| Vitamin A-rich fruits and vegetables | 805 | 68.7 | 68.2 | 61.1 | 66.5 |
| Other vegetables | 805 | 98.0 | 100.0 | 99.7 | 99.5 |
| Other fruits | 805 | 26.7 | 28.6 | 39.2 | 28.9 |
| Values by arm are reported as percentages, based on any consumption over the repeated diet recalls  PLA= Participatory Learning and Action | | | | | |

**Supplemental table 3: Intra-household nutrient allocation ratios, by study arm**

|  | **Mean ± SD (median)**  Outcome on normal scale | | | | |
| --- | --- | --- | --- | --- | --- |
| **Outcome** | ***n*** | **Control** | **PLA** | **PLA + cash** | **PLA + food** |
| **Pregnant women vs household heads** | | | | | |
| Relative Dietary Energy Adeqacy Ratios (RDEARs) ^1^ | 803 | 0.86 ±0.30 (0.83) | 0.79 ±0.30 (0.75) | 0.85 ±0.33 (0.80) | 0.93 ±0.34 (0.86) |
| Relative dietary iron adequacy ratio ^2^ | 803 | 0.40 ±0.18 (0.37) | 0.37 ±0.15 (0.35) | 0.42 ±0.24 (0.38) | 0.44 ±0.18 (0.40) |
| Relative total iron adequacy ratio ^2^ | 803 | 0.70 ±0.82 (0.42) | 0.85 ±0.99 (0.46) | 1.21 ±1.52 (0.61) | 0.94 ±0.93 (0.56) |
| Mean Probability of micronutrient Adequacy (MPA) ratio ^2^ | 801 | 0.68 ±0.41 (0.62) | 0.71 ±0.38 (0.66) | 0.75 ±0.42 (0.72) | 0.70 ±0.37 (0.65) |
| **Pregnant women vs mothers-in-law** | | | | | |
| Relative Dietary Energy Adeqacy Ratios (RDEARs) ^1^ | 799 | 0.91 ±0.31 (0.88) | 0.86 ±0.33 (0.83) | 0.97 ±0.40 (0.88) | 1.03 ±0.40 (0.95) |
| Relative dietary iron adequacy ratio ^2^ | 800 | 0.63 ±0.20 (0.60) | 0.63 ±0.25 (0.60) | 0.71 ±0.34 (0.61) | 0.71 ±0.28 (0.65) |
| Relative total iron adequacy ratio ^2^ | 800 | 1.08 ±1.11 (0.65) | 1.39 ±1.49 (0.81) | 2.08 ±2.33 (1.10) | 1.54 ±1.62 (0.93) |
| Mean Probability of micronutrient Adequacy (MPA) ratio ^2^ | 802 | 0.73 ±0.36 (0.68) | 0.78 ±0.43 (0.71) | 0.80 ±0.47 (0.77) | 0.75 ±0.42 (0.72) |
| Values are reported as mean on normal scale ± SD (median)  PLA= Participatory Learning and Action | | | | | |

**Supplemental table 4: Intra-household food allocation ratios, by study arm**

|  |  | **% pregnant women eating more than the compared household member** | | | |
| --- | --- | --- | --- | --- | --- |
| **Outcome** | ***n*** | **Control** | **PLA** | **PLA+ cash** | **PLA+ food** |
| **Pregnant women vs household heads** | | | | | |
| Flesh foods | 805 | 7.3 | 16.2 | 12.0 | 16.1 |
| Dairy foods | 805 | 27.3 | 26.6 | 36.0 | 32.1 |
| Green leafy vegetables | 805 | 32.0 | 31.2 | 37.1 | 33.5 |
| **Pregnant women vs mothers-in-law** | | | | | |
| Flesh foods | 805 | 14.7 | 20.8 | 20.1 | 21.1 |
| Dairy foods | 805 | 40.7 | 39.6 | 48.8 | 43.1 |
| Green leafy vegetables | 805 | 33.3 | 35.1 | 35.7 | 38.1 |
| Values by arm are reported as percentages^,^ based on average consumption of up to three dietary recalls per person  PLA= Participatory Learning and Action | | | | | |
